# Amelioration of Diabetic Mouse Nephropathy by Catalpol Correlates with Down-Regulation of Grb10 Expression and Activation of Insulin-Like Growth Factor 1 / Insulin-Like Growth Factor 1 Receptor Signaling

**DOI:** 10.1371/journal.pone.0151857

**Published:** 2016-03-17

**Authors:** Shasha Yang, Huacong Deng, Qunzhou Zhang, Jing Xie, Hui Zeng, Xiaolong Jin, Zixi Ling, Qiaoyun Shan, Momo Liu, Yuefei Ma, Juan Tang, Qianping Wei

**Affiliations:** 1 Department of Geriatrics, The First Affiliated Hospital of Chongqing Medical University, Chongqing, China; 2 Department of Endocrinology, The First Affiliated Hospital of Chongqing Medical University, Chongqing, China; 3 Department of Oral Surgery and Pharmacology, School of Dental Medicine, University of Pennsylvania, Philadelphia, Pennsylvania, United States of America; University of Edinburgh, UNITED KINGDOM

## Abstract

Growth factor receptor-bound protein 10 (Grb10) is an adaptor protein that can negatively regulate the insulin-like growth factor 1 receptor (IGF-1R). The IGF1-1R pathway is critical for cell growth and apoptosis and has been implicated in kidney diseases; however, it is still unknown whether Grb10 expression is up-regulated and plays a role in diabetic nephropathy. Catalpol, a major active ingredient of a traditional Chinese medicine, Rehmannia, has been reported to possess anti-inflammatory and anti-aging activities and then used to treat diabetes. Herein, we aimed to assess the therapeutic effect of catalpol on a mouse model diabetic nephropathy and the potential role of Grb10 in the pathogenesis of this diabetes-associated complication. Our results showed that catalpol treatment improved diabetes-associated impaired renal functions and ameliorated pathological changes in kidneys of diabetic mice. We also found that Grb10 expression was significantly elevated in kidneys of diabetic mice as compared with that in non-diabetic mice, while treatment with catalpol significantly abrogated the elevated Grb10 expression in diabetic kidneys. On the contrary, IGF-1 mRNA levels and IGF-1R phosphorylation were significantly higher in kidneys of catalpol-treated diabetic mice than those in non-treated diabetic mice. Our results suggest that elevated Grb10 expression may play an important role in the pathogenesis of diabetic nephropathy through suppressing IGF-1/IGF-1R signaling pathway, which might be a potential molecular target of catalpol for the treatment of this diabetic complication.

## Introduction

Diabetic nephropathy (DN) is one of the major causes of the late stage of renal diseases worldwide, and >25% of patients with Type 1 and 2 diabetes suffer from DN. DN not only seriously affects the health and quality of life of patients but also places a major burden on healthcare resources. [1–3]

Growth factor receptor-binding protein 10 (Grb10) is a member of the adaptor protein superfamily. [4] In humans, the Grb10 gene is located on chromosome 7p11.2–12 [5], and was first cloned in 1995. [6] The regulatory functions of Grb10 have been studied both *in vitro* and *in vivo*, which has been implicated in the regulation of cell growth, proliferation, and plasma membrane translocation of glucose transporter 4 (GLUT4). [7,8] It has been reported that Grb10 inhibits apoptosis through interacting with Bim L.[9] A disruption of the imprinted Grb10 gene in mice exhibited altered body composition, glucose homeostasis, and insulin signaling during postnatal life and could promote tumor formation. [10,11] Using gain-of-function or loss-of-function approaches, several studies have demonstrated that Grb10 functions to negatively regulate IGF-1-mediated signaling both *in vitro* and *in vivo*. [12,13] In diabetic neuropathy, knockdown of Grb10 expression could improve cognitive disorders. [14] In skeletal muscles, Grb10 regulates the development and number of muscle fibers. [15] Meanwhile, Grb10 has been shown to play an important role in regulating islet function and β-cell apoptosis. [16,17] However, it is unclear whether Grb10 is involved in the development of diabetic nephropathy.

Catalpol is an iridoid glucoside extracted from the plant *Rehmannia glutinosa*. *R*. *glutinosa*, a traditional Chinese medicine, and has long been used to treat diabetes in China. Both *in vitro* and *in vivo* studies have reported that catalpol exerts important and extensive pharmacological activities, including anti-inflammatory, anti-aging, and anti-apoptosis activities.[18–20] Compelling evidence has indicated that catalpol exhibits protective effects against oxidative stress, inflammation, and subsequent tissue injuries associated with various diabetic complications, including diabetic nephropathy. [21,22]

In this study, we observed the effect of catalpol on kidney pathology and dysregulated renal functions in streptozotocin (STZ)-induced diabetic mice. Our results indicate that catalpol treatment improved renal functions and ameliorated pathological changes and concomitantly down-regulated Grb10 expression in kidneys of diabetic mice. Additionally, catalpol-induced down-regulation of Grb10 expression correlated with up-regulation of IGF-1 mRNA expression and IGF-1R phosphorylation in kidneys of diabetic mice. These findings suggest that elevated Grb10 expression may contribute to diabetic nephropathy via suppressing IGF-1/IGF-1R signaling pathways, thus serving a potential molecular target of catalpol for the treatment of diabetic nephropathy.

## Materials and Methods

### Ethics statement

This study was performed according to the International Guiding Principles for Biomedical Research Involving Animals of the Council for International Organizations of Medical Sciences. Animal experiments were approved by the Chongqing Medical University Committee on the Ethics of Animal Experiments (Permit Number: 2012–0001). All animal procedures were performed under sodium pentobarbital anesthesia, and all efforts were made to minimize the suffering.

### Animal models

A total of 35 male C57BL/6 mice (6–7 weeks old, weighing 20–22 g) were purchased from the Experimental Animal Center of Chongqing Medical University (Chongqing, China) and housed in a specific pathogen free Laboratory Animal Room (21°C ± 2°C, 12/12 h day/night cycle, with lights on at 08:00). Throughout the experiment, mice were provided free access to food and water. After 1 week, 25 mice were randomly selected to receive a single injection of 180 mg/kg STZ (Sigma-Aldrich, USA). STZ was dissolved in 0.1-M sodium citrate-hydrochloric acid buffer solution (pH 4.5). The remaining mice [the control group (Con)] were injected with an equal volume of buffer solution. Metabolic cages were used to collect the urine of mice, while blood samples were obtained from the tail vein of mice, and blood glucose level was measured using a glucometer (Accu-Check Aviva, Roche Diagnostics, Basel, Germany). Animals with a blood glucose level >16.7 mmol/l at 72 h after STZ injection were considered to be diabetic. [23] The diabetic mice were further randomly divided into two groups, the diabetes mellitus (DM) group and DM treated with catalpol (DM + Cat) group (n = 10 per group).

### Chemical characteristics and source of catalpol

Catalpol is an iridoid glucoside extracted from the plant *R*. *glutinosa*, and its molecular formula is C_15_H_22_O_10_ and molecular weight is 362.33 g/mol. [22] Catalpol (purity 98%) was obtained from Shanghai PureOne Biotechnology (Shanghai, China). It was dissolved in normal saline (NS) before the experiment. Eight weeks later, the DM + Cat group mice were treated by intraperitoneal (i.p) injection of catalpol (10 mg/kg/day) for 14 consecutive days. [24] DM and control group mice were injected with an equal volume of normal saline (NS).

### Kidney weight index and measurement of the 24-h urinary protein excretion, blood urea nitrogen, and serum creatinine

The kidney weight index (KWI, the ratio of kidney weight to body weight) was recorded as a measure of kidney hypertrophy. Urinary protein excretion (UPE) was measured over 24 h using a Bradford protein quantitative assay (Nanjing Keygen Biotech CO. LTD, Nanjing, China). Blood urea nitrogen (BUN) and serum creatinine were measured using and Urea Assay Kit and a Creatinine Assay Kit, respectively (Nanjing Jiancheng Bioengineering Institute, Nanjing, China). All experiments were performed according to the manufacturers’ protocols.

### Periodic acid-Schiff staining, Masson staining and Immunohistochemistry

Animals were anesthetized by intraperitoneal injection of 1% pentobarbital solution and then transcardially perfused with 0.9% sodium chloride solution. The right kidneys were removed and immersed in 4% paraformaldehyde for 24 h. After dehydration, tissues were paraffin-embedded and 4–5 μm sections were cut by using a Paraffin slicer. paraffin sections were dewaxed by baking in the oven at 60°C and soaking in xylene and graded alcohol. Following deparaffinization, tissue sections were washed in phosphate buffered saline (PBS), and incubated in 0.3% hydrogen peroxide for 20 min to remove endogenous peroxidase activity. For antigen retrieval, the sections were immersed in 10 mmol/L sodium citrate buffer (pH 6.0–6.3), and heated in the microwave oven for 15 min at approximately 95°C. After washing in PBS, sections were blocked in 5% goat serum and incubated with the primary antibody solution (Grb10 antibody, dilution 1:300, caspase-3, dilution 1:200; Abcam, USA) overnight at 4°C. The next day, sections were rewarmed for 1 h at 37°C. After extensive washing with PBS, tissue sections were incubated with biotinylated goat antirabbit secondary antibody (dilution 1:200) at 37°C for 45 min. After washing with PBS, sections were incubated with horseradish peroxidase-labeled streptavidin solution at 37°C for 30 min and then washed in PBS again. Further, sections were incubated in a 3,30-diaminobenzidine solution (Sigma, USA) until brown staining developed. After extensively washing with water, the nuclei were counter-stained with hematoxylin. Finally, tissue sections were mounted with neutral gum. An Olympus PM 20 (Olympus, Tokyo, Japan) was used to photograph the sections. Image-Pro Plus, version 6.0 (Media Cybernetics, Inc., Silver Spring, MD, USA) was used to measure the average optical density under the same conditions.

### Quantitative polymerase chain reaction

RNeasy Mini kit (Qiagen, Mississauga, ON, Canada) was used to extract total RNA from the kidney cortex tissues. The concentration and purity of total RNA was measured using a NanoDrop 2000c (Thermo Fisher Scientific Inc., Waltham, MA, USA). The pure RNA had an A260/A280 ratio of 1.8 and 2.0. cDNA was synthesized from mRNA using the PrimeScript RT Reagent kit (Takara Bio Inc.,Otsu, Japan) and the same machine was used to detect the purity and concentration of cDNA. The primers of target genes were designed and synthesized by Sangon Biotech (Shanghai, China). Primer sequences were as follows: Grb10, forward, 5’-GTGAAAGAGGAGGACGCAAGT-3’;reverse: 5’-TCCAGCAATCAGGTAGAAGATG-3’; IGF-1, forward, 5’-AAGGCAGTTTACCCAGGCTC-3’; reverse, 5’-TCTTTATTGCAGGTGCGGTCA-3’; β-actin, forward, 5’-GTGCTATGTTGCTCTAGACTTCG-3’; reverse, 5’-ATGCCACAGGATTCCATACC-3’. The following components were added to reaction tubes: 12.5 μl of SYBR Premix Ex Taq (Tli RNaseH Plus), 1.0 μl of forward primer, 1.0 μl reverse primer, 200 ng of cDNA template, and 8.5 μl of dH_2_O. Then, the contents were amplified under the following conditions: 95.0°C for 30 sec, 40 cycles of 95.0°C for 5 sec, and 60.0°C for 30 sec, with the melt curve at 65.0–95.0°C. The comparative threshold cycle (Ct) for quantitative target gene expression associated with β-actin was analyzed using Bio-Rad CFX Manager software (Bio-Rad, Hercules, CA, USA). The relative change of gene expression was calculated using the 2^-DDCt^ equation.

### Western blot analysis

The kidney cortex tissues were completely polished with tissue total protein lysis buffer. Tissue total protein lysis buffer was produced by mixing RIPA, PMSF, and phosphatase inhibitors in certain proportions. Protein concentration was estimated using a bicinchoninic acid (BCA) protein assay kit (Beyotime Institute of Biotechnology, China). Tissue lysates used for immunoprecipitation, or 50–80 μg of protein per sample, were directly analyzed by SDS-PAGE, then transferred to membranes that were probed with the following antibodies: a rabbit polyclonal antibody for Grb10 (1:1500, Abcam, USA), a rabbit polyclonal antibody for IGF-1R (1:1000; ImmunoWay Biotechnology Inc., Newark, DE, USA) and phospho-IGF-1R (Y1161) (1:1000; ImmunoWay Biotechnology Inc., USA), and a mouse monoclonal antibody for β-actin (1:1000; Beijing Zhongshan Golden Bridge Biotechnology Co. Ltd, Beijing, China).

### Statistical analysis

All results were analyzed using SPSS 19.0 (SPSS Inc., Chicago, IL, USA) and presented as the mean ± SEM. One way analysis of variance (one-way ANOVA) was conducted. All statistical tests were two-sided with statistical significance set at p < 0.05.

## Results

### Effect of chronic hyperglycemia and catalpol in mice

Diabetes is caused by defects in insulin secretion and/or action, resulting in chronic hyperglycemia and metabolic diseases. [25] Abnormal metabolism of glucose and lipids is important contributors to the development of complications in diabetes. [26,27] In our study, mice that were intraperitoneally injected with STZ (the DM group) had higher blood glucose levels than control mice without STZ administration. Over time, diabetic mice exhibited symptoms such as polydipsia and polyuria. After a few weeks, several diabetic mice exhibited listlessness and decreased activity. During the experiment, animals with a blood glucose level < 16.7 mmol/l or died were excluded in the experimental study. In the end, six diabetic mice were included in the DM group while seven of them were included in DM group with catalpol treatment.

The mean blood glucose level was significantly higher in DM mice than in non-DM control mice (P < 0.01). After 2 weeks of treatment with catalpol, the level of blood glucose in DM + Cat group had no significant decline in comparison with that in non-treated DM mice (P > 0.05, Table 1). These results imply that a longer treatment time might be required for catalpol to exert an hypoglycemic effect.

**Table 1:** The measurement results of indicators in each group.n = 6,$\bar{x}$±s.

Group	Con	DM	DM+Cat
**Indicator**			
**Glycemia (mmol/l) ①**	6.7833±0.76790	28.0875±0.64240**	27.6375±0.80167
**KWI ②**	0.0072±0.00118	0.0117±0.00163**	0.0101±0.00152^ΔΔ^
**24h urinary protein (mg/24h) ③**	0.0686±0.04449	0.9362±0.42527**	0.4939±0.32543^Δ^
**BUN (mmol/l) ④**	5.4026±1.01644	9.9609±1.35238**	8.1559±0.94499^Δ^
**SCr (μmol/l) ⑤**	56.9184±14.35010	109.0958±19.50298**	92.9539±8.90225^Δ^

Con: normal control group; DM: diabetes mellitus group; DM + Cat: diabetes mellitus treated with catalpol group.

*P < 0.05 vs Con;

**P < 0.01 vs Con

^Δ^P < 0.05 vs DM;

^ΔΔ^P < 0.01 vs DM.

BUN: Blood urea nitrogen; SCr: Serum creatinine.

### Catalpol influenced the renal function

We then determined whether catalpol has a protective effect on renal function in diabetic mice. As shown in Table 1, the renal function of diabetic mice was severely damaged, which was manifested as proteinuria and elevated serum creatinine levels and blood urea nitrogen levels. Following administration with catalpol, the 24 h urinary protein excretion, serum creatinine levels, and blood urea nitrogen in the DM + Cat group was significantly lower than that in the non-treated DM group (P < 0.05). These results suggest that catalpol could significantly improve the impaired renal functions in diabetic mice.

### Expression of endogenous Grb10 in diabetic nephropathy

**Fig 1:**
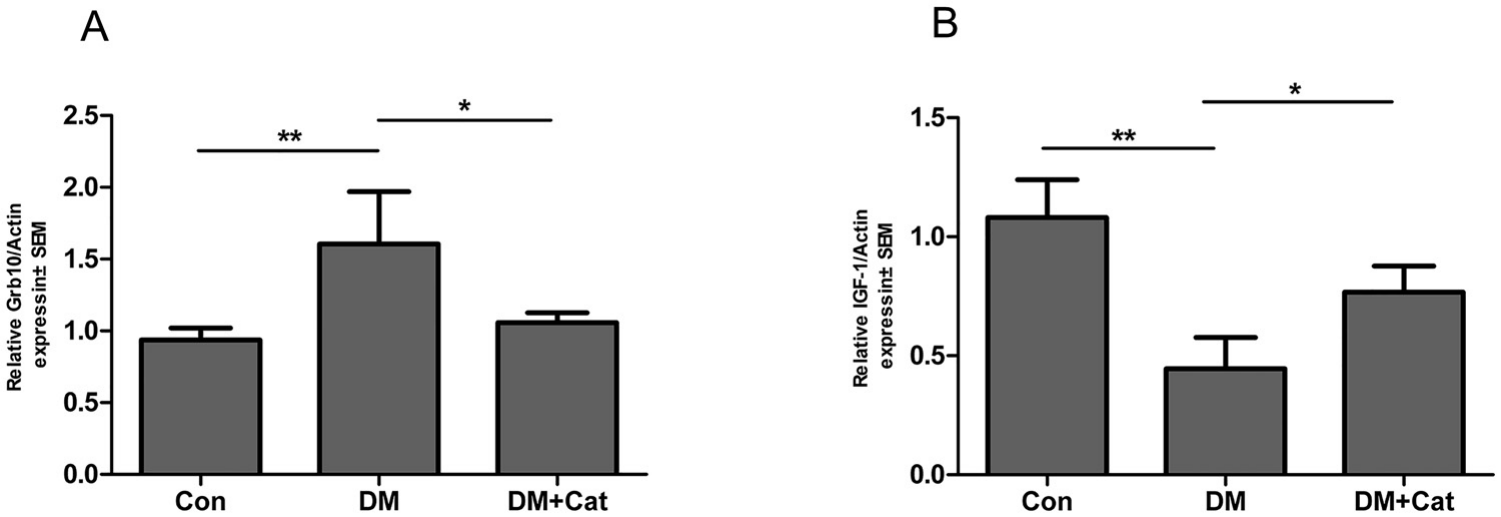
Quantitative real-time PCR analysis of Grb10 and IGF-1 mRNA expressions in kidneys. The levels of Growth factor receptor bound protein 10(Grb10) (A) and insulin-like growth factor 1(IGF- 1) (B) mRNA expressions were measured using qPCR. The average levels were shown in the graphs. The data were shown as the mean ± SEM (A, n = 3; B, n = 5). (*, P < 0.05; **, p < 0.01). Abbreviations: Con: normal control group; DM: diabetes mellitus group; DM + Cat: diabetes mellitus treated with catalpol group.

**Fig 2:**
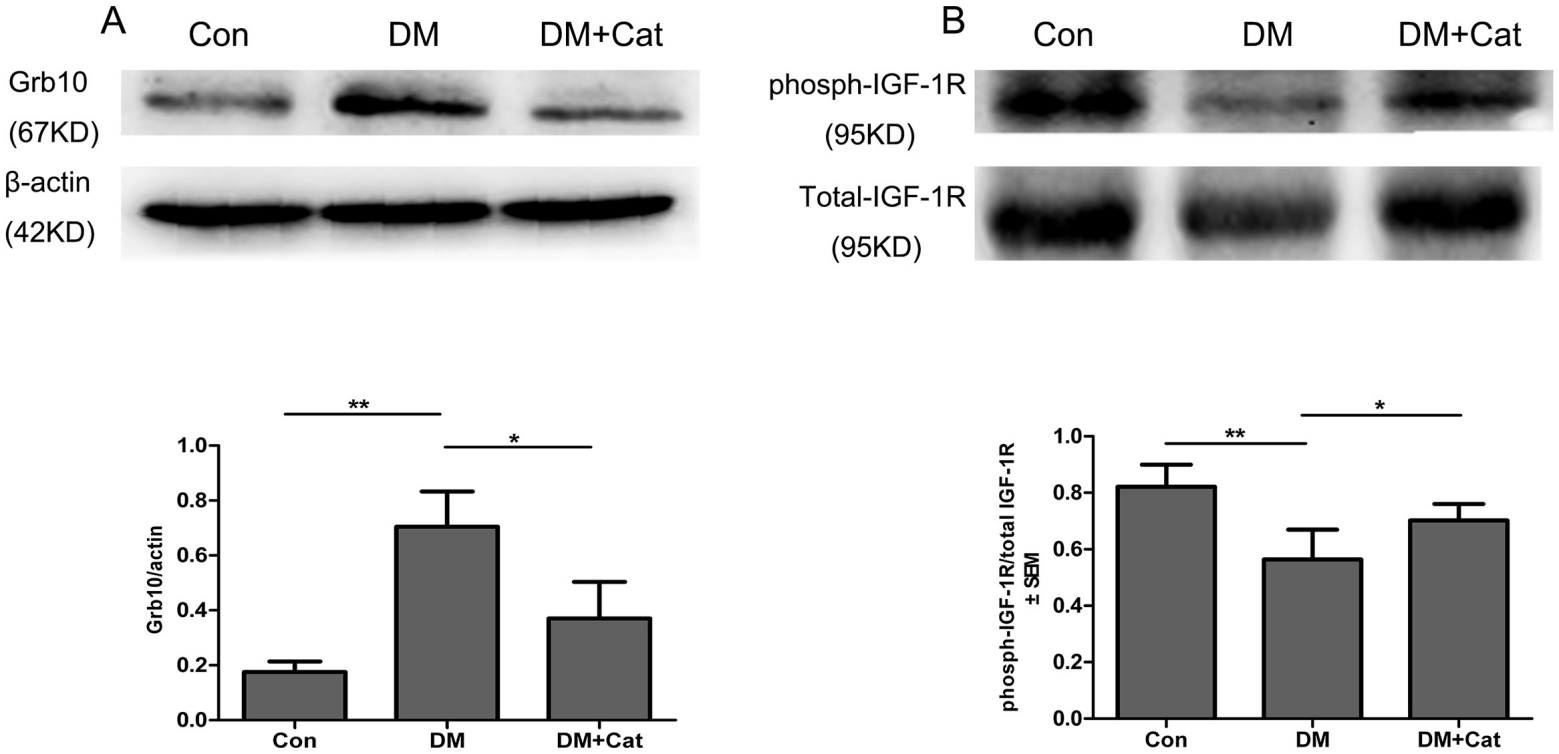
The expression levels of Grb10 and IGF-1R proteins in kidneys. The levels of Growth factor receptor bound protein 10 (Grb10) (A) and Insulin-like growth factor 1 receptor (IGF- 1R) (B) proteins were measured by Western Blotting. Statistical analysis was done using factorial ANOVA with Fisher’s multiple comparisons post (*, P < 0.05; **, P < 0.01). The data were shown as the mean ± SEM (A, n = 3; B, n = 5). Abbreviations: Con: normal control group; DM: diabetes mellitus group; DM + Cat: diabetes mellitus treated with catalpol group.

Using qPCR and Western blotting, we examined alterations in the expression of Grb10 in the kidney. The expression levels of Grb10 mRNA and protein in the kidney tissues were significantly higher in the DM group than those in non-diabetic control group (Figs 1A and 2A; P < 0.01). On the contrary, Grb10 mRNA and protein levels in the kidney tissues were significantly down-regulated in the DM + Cat group as compared to non-treated DM group (P < 0.05). These results suggest that catalpol-mediated protective effects on diabetic nephropathy correlate with down-regulated Grb10 expression in diabetic kidneys.

### Expression of IGF-1 and IGF-1R in the kidney

Since Grb10 has been demonstrated to negatively regulate IGF-1/IGF-1R signaling,[12] we then determined whether catalpol-mediated down-regulation of Grb10 expression was coupled to up-regulation of IGF-1/IGF-1R signaling in diabetic kidneys. As shown in Fig 1B, the IGF-1 mRNA levels were lower in the kidney tissues of DM mice than those in non-diabetic control mice (P < 0.01). Consistently, the level of IGF-1R phosphorylation (phosph-IGF-1R, the phosphorylation site of Tyr1161) was also significantly lower in the DM group than that in the non-diabetic control group (Fig 2B; P < 0.01). Additionally, IGF-1 mRNA levels and IGF-1R phosphorylation were higher in kidneys of the DM + Cat group than those in the non-treated DM group (P < 0.05). These findings suggest that the elevated Grb10 expression was reversely associated with a lower level of IGF-1/IGF-1R signaling in diabetic kidneys, thus might play an important role in diabetic nephropathy.

### Periodic acid-Schiff, Masson and immunohistochemical staining of Kidney Tissues

**Fig 3:**
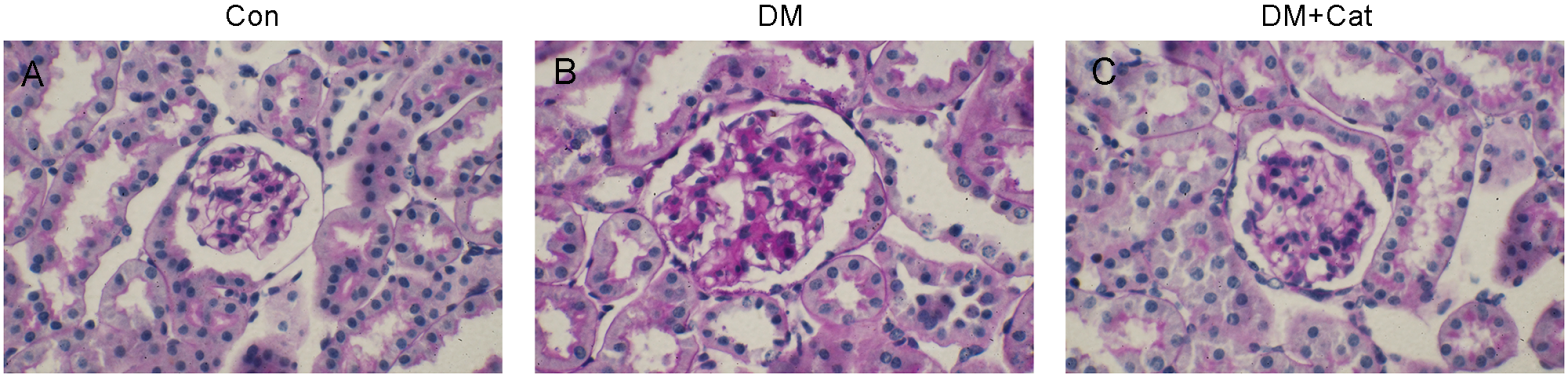
Periodic acid-Schiff staining of kidney tissues in each group. The kidney tissues were stained with Periodic acid-Schiff. Cells with a positive reaction were stained purple (magnification, ×400). In picture B, fuchsia positive cells were more than that of the other two groups. A (Con): normal control group; B (DM): diabetes mellitus group; C (DM + Cat): diabetes mellitus treated with catalpol group.

**Fig 4:**
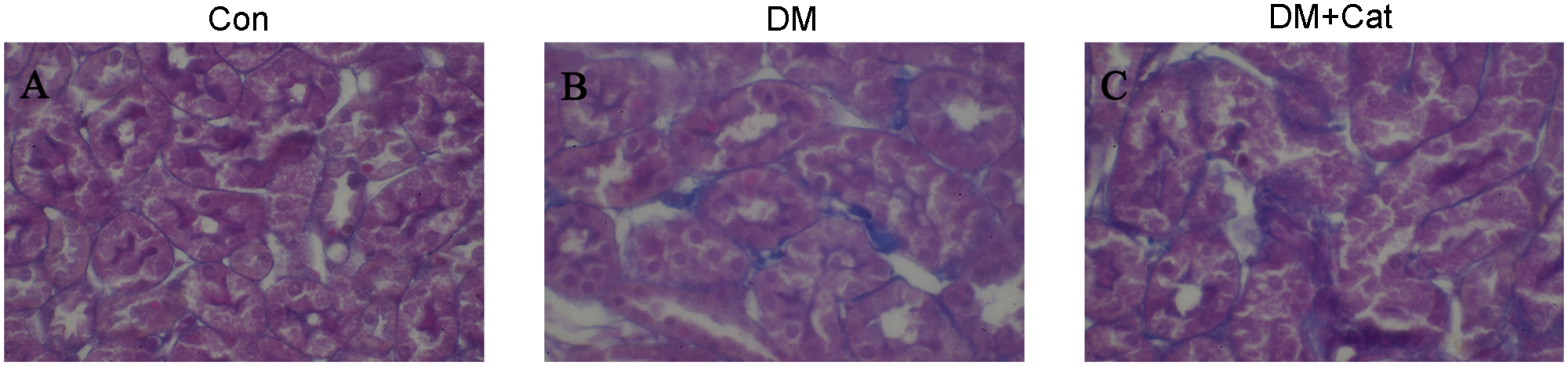
Masson staining of kidney tissues in each group. The topically microscopic images of the kidney tissues were stained with ponceau and aniline blue dye. Positive reaction is represented by blue staining (magnification, x400). In picture B, blue stained areas were more than that of the other two groups, indicating more severe fibrosis. A (Con): normal control group; B (DM): diabetes mellitus group; C (DM + Cat): diabetes mellitus treated with catalpol group.

Glycoproteins are stained purple by Periodic acid-Schiff (PAS) staining. As shown in Fig 3, pathological changes were observed in both DM and DM + Cat groups. However, the degree of staining, representative of pathological severity, was lower in the DM + Cat group than that in the DM group. In addition, in Fig 4, the kidney fibrosis observed in DM + Cat group was significantly attenuated as compared with that in the non-treated DM group, suggesting that catalpol treatment could inhibit diabetic nephrology-related kidney fibrosis.

**Fig 5:**
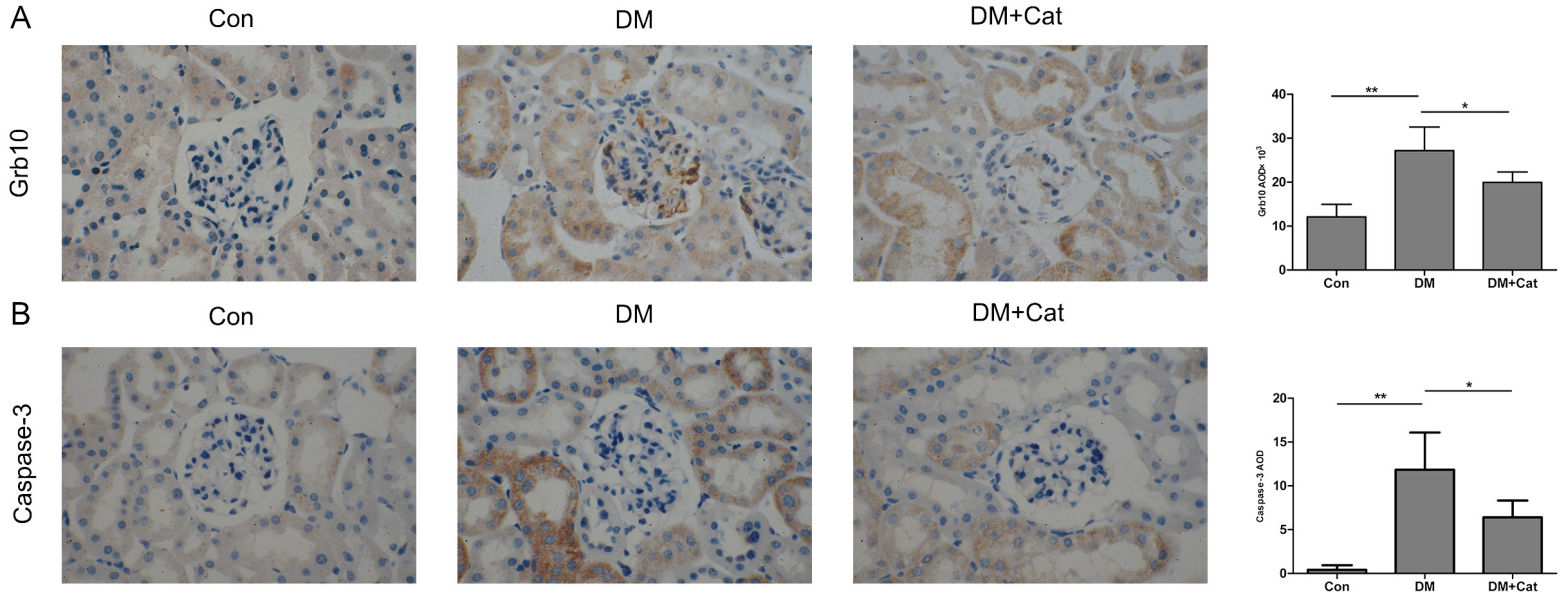
Immunohistochemical study on the expression of Grb10 and caspase-3 proteins in kidneys. The distribution and expression levels of growth factor receptor-bound protein 10 (Grb10) (A) and caspase-3 (B) were determined by immunohistochemical staining. Images shown at magnifications of ×400. Brown granules represent positive results. Average optical density values were measured by Image-Pro Plus, version 6.0, and expressed as the mean ± SEM (n = 4) of two independent experiments. Statistical analysis was performed using factorial analysis of variance with Fisher’s multiple comparisons. (*, p < 0.05; **, p < 0.01). Con: normal control group; DM: diabetes mellitus group; DM + Cat: diabetes mellitus treated with catalpol group.

We also examined the changes in Grb10 protein expression in mouse kidneys at 10 weeks following the induction of diabetes. As shown in Fig 5A, Grb10 protein expression, located both on the cell membrane and in the cytoplasm, was mainly distributed in the glomerulus, tubules and interstitial blood vessels. The expression level of Grb10 protein in kidneys of the DM group was significantly higher than that in the DM + Cat group (p < 0.05). As shown in Fig 5B, caspase-3 expression was increased in kidneys of non-treated diabetic mice, but significantly decreased in kidneys of catalpol-treated diabetic mice (P < 0.05).

## Discussion

Chronic hyperglycemia may cause structural and functional changes in the kidney. Abnormal glucose metabolism can cause a series of pathophysiological changes and simultaneously cause abnormal lipid metabolism. [26] Thus, chronic hyperglycemia causes abnormal metabolism. Intervention studies have convincingly demonstrated that hyperglycemia is a major pathogenic factor for development of various diabetic complications. [28] In the current study, we observed that mice with continuous hyperglycemia developed diabetic nephrology (DN) which was manifested by pathological changes in kidneys, proteinuria, elevated serum creatinine and blood urea nitrogen levels.

### Catalpol ameliorated diabetic nephropathy

Both *in vitro* and *in vivo* studies have indicated that catalpol can exert various pharmacological activities. For example, catalpol is reported to delay cellular senescence and protect against apoptosis. [29] Furthermore, catalpol exhibits anti-inflammatory properties [30] and mitigates diabetic nephropathy by reducing the deposition of extracellular matrix proteins. [22] However, the mechanisms underlying catalpol-mediated beneficial effects on diabetic nephropathy are still unclear. In this study, we showed that administration of catalpol could reverse, to certain degrees, the impaired renal functions and pathological changes in diabetic kidneys. Clinically, proteinuria, serum creatinine and blood urea nitrogen are commonly used to evaluate renal functions, which are gradually elevated upon the impairment of renal functions. [31] Catalpol treatment decreased the 24 h-urinary protein excretion, serum creatinine and blood urea nitrogen levels. These observations suggest that catalpol can protect against diabetic nephropathy by ameliorating renal function loss.

Apoptosis of kidney cells is a hallmark in diabetic nephropathy fibrosis. Caspase-3 is a crucial executor or initiating factor of cell apoptosis. [32,33] Histologically, we demonstrated that fibrosis developed in the kidney tissues of diabetic mice. During chronic hyperglycemia, kidney cell apoptosis appeared to be initiated. We observed that Catalpol administration significantly decreased caspase-3 expression, suggesting that catalpol could inhibit caspase3-mediated apoptosis in DN. Therefore, we speculated that catalpol ameliorated DN-associated kidney fibrosis possibly by protecting renal cells from apoptosis. In addition, our data showed improvement in DM-associated abnormal structures of the glomerulus and tubules following catalpol treatment, suggesting that catalpol can also protect against pathological damages in DN.

### Elevated Grb10 expression may play a role in diabetic nephropathy but could be reversed by catalpol

Grb10 has also been shown to be involved in regulating insulin signaling pathways and metabolic actions. [34,35] Here, we examined the potential role of Grb10 in diabetic nephropathy and have revealed that Grb10 protein expression was located in both the glomerulus and tubules and increased along with the development and progression of diabetic nephropathy. The increased expression of Grb10 was correlated with renal function impairment and pathological changes in kidneys of diabetic mice. Thus, elevation of Grb10 expression may have a detrimental effect on the development and progression of DN. Meanwhile, we found that catalpol treatment significantly abrogated the elevated expression of Grb10 protein in diabetic kidneys, suggesting that catalpol ameliorated renal function loss and pathological damages possibly by down-regulating Grb10 expression in diabetic nephropathy.

### Elevated Grb10 expression correlates with decreased levels of IGF-1/IGF-1R signaling in diabetic nephropathy

Grb10, an adaptor protein, binds to tyrosine-phosphorylated receptor, IGF-1R, and subsequently inhibits IGF-I signaling.[36] It has been shown that Grb10 inhibits IGF-1/IGF-1R signaling via blocking the access of phosphatase to the activated IGF-I receptor.[12] As is known, IGF-1 exerts its biological functions via activating IGF-1R-mediated downstream signaling pathways. Previous study showed that IGF-1 can inhibit mesangial cell apoptosis and hyperglycemia-induced DNA damage and promote DNA repair via activating IGF-1R signaling. [37,38] Aberrant IGF-1/IGF-1R signaling has been implicated in various kidney diseases, [39,40] suggesting that IGF-1/IGF-1R may play a vital role in the development of diabetic nephropathy by regulating cell growth and apoptosis. In the current study, we found that an elevated expression of Grb10 correlated with a decreased level of IGF-1/IGF-1R signaling in diabetic kidneys, while treatment of diabetic mice with capaltol not only down-regulated Grb10 expression but also simultaneously up-regulated IGF-1 mRNA expression and IGF-1R phosphorylation in diabetic kidneys. Collectively, our findings support the notion that catalpol exerts its therapeutic effects on diabetic nephropathy possibly by down-regulating Grb10 expression and the subsequent activation of IGF-1/IGF-1R signaling.

## Conclusion

In conclusion, our data indicates that continuous hyperglycemia might lead to an elevated expression of Grb10 and the subsequent decrease in IGF-1/IGF-1R signaling in kidneys, which may consequently contribute to the pathogenesis of diabetic nephropathy. Catalpol treatment could improve diabetes-associated impaired renal functions and ameliorate pathological changes in diabetic kidneys, while such beneficial effects correlate with a down-regulation of Grb10 expression and a concomitant up-regulation of IGF-1/IGF-1R signaling in diabetic kidneys. These findings suggest that elevated Grb10 expression may contribute to diabetic nephropathy via suppressing IGF-1/IGF-1R signaling pathways, thus serving a potential molecular target of catalpol for the treatment of diabetic nephropathy. But more studies are warranted for further mechanistic exploration.

## Supporting Information

S1 Table The number of animals in each group. Con: normal control group; DM: diabetes mellitus group; DM +Cat: diabetes mellitus treated with Catalpol group.
(PDF)

S2 Table Results of Fig 1A. P (P1,P2) present “Significance”. The significant difference between the mean level of 0.05. P<0.05 means the results are statistically significant. **, DM vs Con or Con vs DM; *, DM vs DM+Cat or DM+Cat vs DM:^Δ^,Con vs DM+Cat or DM+Cat vs Con. Con: normal control group; DM: diabetes mellitus group; DM +Cat: diabetes mellitus treated with Catalpol group.
(PDF)

S3 Table Results of Fig 1B. (PDF)

S4 Table Results of Fig 2A. (PDF)

S5 Table Results of Fig 2B. (PDF)

S6 Table Results of Fig 5A. (PDF)

S7 Table Results of Fig 5B. (PDF)
