# Supplementary material for: Amelioration of Diabetic Mouse Nephropathy by Catalpol Correlates with Down-Regulation of Grb10 Expression and Activation of Insulin-Like Growth Factor 1 / Insulin-Like Growth Factor 1 Receptor Signaling
Source: PLoS One. 2016 Mar 17;11(3):e0151857. doi: 10.1371/journal.pone.0151857 (PMC4795681; doi:10.1371/journal.pone.0151857)
Supplement: S1 Table — Con: normal control group; DM: diabetes mellitus group; DM +Cat: diabetes mellitus treated with Catalpol group. (PDF) [file pone.0151857.s001.pdf]

S1 Table. The number of animals in each group

Group	Con	DM	DM+Cat
Content			
The number of packets ①	10	10	10
The number of successful model animals ②	10	6	7
The number of animals to restore blood sugar ③	0	1	1
The number of died animals ④	0	3	2

Con: normal control group; DM: diabetes mellitus group; DM +Cat: diabetes mellitus treated with Catalpol group.
