# Supplementary material for: Amelioration of Diabetic Mouse Nephropathy by Catalpol Correlates with Down-Regulation of Grb10 Expression and Activation of Insulin-Like Growth Factor 1 / Insulin-Like Growth Factor 1 Receptor Signaling
Source: PLoS One. 2016 Mar 17;11(3):e0151857. doi: 10.1371/journal.pone.0151857 (PMC4795681; doi:10.1371/journal.pone.0151857)
Supplement: S2 Table — P (P1,P2) present “Significance”. The significant difference between the mean level of 0.05. P<0.05 means the results are statistically significant. **, DM vs Con or Con vs DM; *, DM vs DM+Cat or DM+Cat vs DM:Δ,Con vs DM+Cat or DM+Cat vs Con. Con: normal control group; DM: diabetes mellitus group; DM +Cat: diabetes mellitus treated with Catalpol group. (PDF) [file pone.0151857.s002.pdf]

S2 Table. Results of Fig 1A

Result	Mean	Standard deviation	P1	P2
**Group**				
**Con**	0.9361	0.08406	0.010**	0.528$^{\Delta}$
**DM**	1.6058	0.36483	0.010**	0.022*
**DM+Cat**	1.0563	0.07027	0.528$^{\Delta}$	0.022*

P (P1,P2) present “Significance”. The significant difference between the mean level of 0.05. P<0.05 means the results are statistically significant. **, DM vs Con or Con vs DM ;*, DM vs DM+Cat or DM+Cat vs DM: $^{\Delta}$,Con vs DM+Cat or DM+Cat vs Con . Con: normal control group; DM: diabetes mellitus group; DM +Cat: diabetes mellitus treated with Catalpol group.
