# Supplementary material for: Amelioration of Diabetic Mouse Nephropathy by Catalpol Correlates with Down-Regulation of Grb10 Expression and Activation of Insulin-Like Growth Factor 1 / Insulin-Like Growth Factor 1 Receptor Signaling
Source: PLoS One. 2016 Mar 17;11(3):e0151857. doi: 10.1371/journal.pone.0151857 (PMC4795681; doi:10.1371/journal.pone.0151857)
Supplement: S3 Table — (PDF) [file pone.0151857.s003.pdf]

S3 Table. Results of Fig 1B

Result	Mean	Standard deviation	P1	P2
Group				
Con	0.8210	0.07839	<0.001**	0.043$^{\Delta}$
DM	0.5641	0.10588	<0.001**	0.022*
DM+Cat	0.7021	0.05808	0.043$^{\Delta}$	0.022*

P (P1,P2) present "Significance". The significant difference between the mean level of 0.05. P<0.05 means the results are statistically significant. **, DM vs Con or Con vs DM ;*, DM vs DM+Cat or DM+Cat vs DM: $^{\Delta}$,Con vs DM+Cat or DM+Cat vs Con . Con: normal control group; DM: diabetes mellitus group; DM +Cat: diabetes mellitus treated with Catalpol group.
