# Supplementary material for: Amelioration of Diabetic Mouse Nephropathy by Catalpol Correlates with Down-Regulation of Grb10 Expression and Activation of Insulin-Like Growth Factor 1 / Insulin-Like Growth Factor 1 Receptor Signaling
Source: PLoS One. 2016 Mar 17;11(3):e0151857. doi: 10.1371/journal.pone.0151857 (PMC4795681; doi:10.1371/journal.pone.0151857)
Supplement: S4 Table — (PDF) [file pone.0151857.s004.pdf]

S4 Table. Results of Fig 2A

**Result**	**Mean**	**Standard deviation**	**P1**	**P2**
**Group**				
**Con**	0.1758	0.03824	0.001**	0.071$^{\Delta}$
**DM**	0.7045	0.12859	0.001**	0.010*
**DM+Cat**	0.3708	0.13302	0.071$^{\Delta}$	0.010*

P (P1,P2) present "Significance". The significant difference between the mean level of 0.05. P<0.05 means the results are statistically significant. **, DM vs Con or Con vs DM ;*, DM vs DM+Cat or DM+Cat vs DM: $^{\Delta}$,Con vs DM+Cat or DM+Cat vs Con . Con: normal control group; DM: diabetes mellitus group; DM +Cat: diabetes mellitus treated with Catalpol group.
