# Supplementary material for: Amelioration of Diabetic Mouse Nephropathy by Catalpol Correlates with Down-Regulation of Grb10 Expression and Activation of Insulin-Like Growth Factor 1 / Insulin-Like Growth Factor 1 Receptor Signaling
Source: PLoS One. 2016 Mar 17;11(3):e0151857. doi: 10.1371/journal.pone.0151857 (PMC4795681; doi:10.1371/journal.pone.0151857)
Supplement: S5 Table — (PDF) [file pone.0151857.s005.pdf]

S5 Table. Results of Fig 2B

Result	Mean	Standard deviation	P1	P2
**Group**				
**Con**	1.0805	0.15942	<0.001**	0.003$^{\Delta}$
**DM**	0.4453	0.13126	<0.001**	0.003*
**DM+Cat**	0.7664	0.11028	0.003$^{\Delta}$	0.003*

P (P1,P2) present "Significance". The significant difference between the mean level of 0.05. P<0.05 means the results are statistically significant. **, DM vs Con or Con vs DM ;*, DM vs DM+Cat or DM+Cat vs DM: $^{\Delta}$,Con vs DM+Cat or DM+Cat vs Con . Con: normal control group; DM: diabetes mellitus group; DM +Cat: diabetes mellitus treated with Catalpol group.
